# A Suggestion or Two

**Published:** 1897-06

**Authors:** Chas. W. Jenkins

**Affiliations:** Zurich, Switzerland


### A Suggestion or Two.

CHAS. W. JENKINS, D.D.S., ZURICH, SWITZERLAND.

At this twenty-first annual meeting of our Society, it may not be an ill-timed question if we ask, what better organized methods of co-operation can help us to serve the public in future with distinction? In a paper which I had the honor of reading to you last year, the service hitherto rendered by Americans to Dentistry in Europe was described as consisting chiefly in raising the general standard of work by utilizing the discoveries of science without regard to time, trouble, or expense, and without yielding to merely speculative theories. This elevation of the standard has been accomplished almost exclusively by painstaking office work rather than by the invention of new methods. Nor has this accomplishment been less positive and real on that account. If our revered Abbot benefitted thousands by demenstrating that the combination of tin and gold has a preservative quality superior to gold or the plastics in certain cases, he rendered a service immeasurably greater by adhering to the practice of using it in the face of professional and lay prejudice and patiently appealing to results, when more brilliant work would have attracted immediate admiration. It is not given to every man to make discoveries; but it is within the power of all properly trained dentists to test carefully, without prejudice and without ill-balanced enthusiasm for novelty, whatever methods, materials, instruments, or principles may be introduced by ingenious and skilful operators. The genius of one may invent; but the talent of many is needed to establish an improvement.

But the comparison of experience in dealing with the multitude of suggested improvements is less open to us in Europe, who are fewer in number and more widely scattered than are the members of our home societies. We meet but once a year; this infrequency of sessions, instead of making it easier, as might be supposed, to fill an annual programme, makes it more difficult. There is material in abundance; what is wanted is enthusiasm and system in bringing it to the fore. Professional enthusiasm grows from the frequent contact of those interested in professional work, since we cannot meet monthly, all of us, how can we otherwise come into closer touch and be more helpful to each other?

Something can be done by local clubs. Indeed a very small dental club has certain special advantages. Less time is lost in formalities; the interchange of views is freer; cases are discussed practically while they are fresh in hand the small details, often of vital importance, are not so likely to be omitted or hurried over half understood as in a larger company. Personal contact and the conversational comparison of methods are more suggestive and stimulating than the reading of papers, however able and comprehensive. We get down to work when we sit elbow to elbow in a small room within reach of tools and beset by no possibility of boring some portion of a crowd. Three live dentists can hardly fail to gain, by frequent conference, in that increased alertness which comes of attention drawn to other men's ideas, whether the ideas be endorsed or not. It is my belief that if the members of this society would meet for professional conference in groups of five or six, they would soon find the sacrifice of the few hours necessary for a short journey and evening session fruitful in the more thoughtful work of their own hands.

Such a local co-operation would also make it easier to secure contributions to our annual programme. The circular is a very poor seed, as concerns sprouting, as all who have acted as secretaries or on the Executive Committee well know. But if those members who now feel the presentation of a paper a great burden were accustomed to present informally the current practical problems of their offices to a smaller company, our meetings would become more fruitful at cost of little special preparation.

In this connection I would also make the suggestion that a more definite and systematized division of responsibility in the furnishing of reports might promote better results. The volunteer paper or clinic should be a report of progress. It is often only a remastication of "chestnuts." We rehear the old story or witness for the hundredth time the old tricks. Some members are loth to tell the story or repeat the tricks, and because they have no grand discovery to exhibit or no new theory to advance, sit silent, chewing the cud of contemptuous criticism. But suppose that each member enter his name on a list kept by the secretary, engaging to report verbally, or by letter if he cannot be present, upon the progress made during the year in the discovery, adaptation or improvements in some narrowly restricted department of our work. To illustrate: The use of separators, the action of newly introduced disinfectants, inlay fillings, hyperdemic injections for extraction, the amputation of pulps, etc;—the object to be, not the history of dentistry since the eruption of the tooth of time, but a collection of facts gathered from current experience. No member need confine his contribution to this report, but if, instead of everything being left to haphazard, each would assume a definite. small responsibility, the ground could be more thoroughly and easily covered.

In conclusion, I venture to call attention to the desirability of more frequent interchange of records or notes on especially obscure and unfinished cases. More than any other class of dentists we Americans in Europe have to deal with a fluctuating clientele. Tourists of all civilized nations are passing from one of us to another, many of them the unenviable posessors of chronic troubles which need treatment but which require more time to understand than is usually at the disposal of one practicioner. The report of a very intelligent patient is apt to be partial and misleading, and ought, whenever possible, to be supplemented by a professional one. In some cases the patient might be trusted with advantage with a written statement of the treatment, to be shown to the operator to whom he is recommended; or a circular letter, sent to the three or four men of the region where he is to spend the coming months, would prevent some of the mistakes and waste of time to which we are all exposed. For one I cannot be too thankful for the kind consideration shown both to my patients and myself whenever a fellow-dentists has taken the time and trouble to send me the history of a case which presents special or obscure conditions.

---

USE other peoples brains all you can, but use your own more energetically than those of others and then you can use theirs to better advantage.—*Love's Lance.*

